# A 2D/3D image analysis system to track fluorescently labeled structures in rod-shaped cells: application to measure spindle pole asymmetry during mitosis

**DOI:** 10.1186/1747-1028-8-6

**Published:** 2013-04-27

**Authors:** Daniel Schmitter, Paulina Wachowicz, Daniel Sage, Anastasia Chasapi, Ioannis Xenarios, Simanis, Michael Unser

**Affiliations:** 1 Biomedical Imaging Group, Ecole Polytechnique Fédérale de Lausanne (EPFL), Lausanne, Switzerland; 2 Cell Cycle Control Laboratory, Swiss Institute for Experimental Cancer Research (ISREC), Ecole Polytechnique Fédérale de Lausanne(EPFL), Lausanne, Switzerland; 3 Swiss-Prot Group & Vital-IT Group, Swiss Institute of Bioinformatics (SIB) Lausanne, Switzerland

**Keywords:** Cell segmentation, Protein tracking, Rod shape, Kymograph, Asymmetry, Fluorescence time-lapse microscopy

## Abstract

**Background:**

The yeast *Schizosaccharomyces pombe* is frequently used as a model for studying the cell cycle. The cells are rod-shaped and divide by medial fission. The process of cell division, or cytokinesis, is controlled by a network of signaling proteins called the Septation Initiation Network (SIN); SIN proteins associate with the SPBs during nuclear division (mitosis). Some SIN proteins associate with both SPBs early in mitosis, and then display strongly asymmetric signal intensity at the SPBs in late mitosis, just before cytokinesis. This asymmetry is thought to be important for correct regulation of SIN signaling, and coordination of cytokinesis and mitosis. In order to study the dynamics of organelles or large protein complexes such as the spindle pole body (SPB), which have been labeled with a fluorescent protein tag in living cells, a number of the image analysis problems must be solved; the cell outline must be detected automatically, and the position and signal intensity associated with the structures of interest within the cell must be determined.

**Results:**

We present a new 2D and 3D image analysis system that permits versatile and robust analysis of motile, fluorescently labeled structures in rod-shaped cells. We have designed an image analysis system that we have implemented as a user-friendly software package allowing the fast and robust image-analysis of large numbers of rod-shaped cells. We have developed new robust algorithms, which we combined with existing methodologies to facilitate fast and accurate analysis. Our software permits the detection and segmentation of rod-shaped cells in either static or dynamic (i.e. time lapse) multi-channel images. It enables tracking of two structures (for example SPBs) in two different image channels. For 2D or 3D static images, the locations of the structures are identified, and then intensity values are extracted together with several quantitative parameters, such as length, width, cell orientation, background fluorescence and the distance between the structures of interest. Furthermore, two kinds of kymographs of the tracked structures can be established, one representing the migration with respect to their relative position, the other representing their individual trajectories inside the cell. This software package, called “RodCellJ”, allowed us to analyze a large number of *S. pombe* cells to understand the rules that govern SIN protein asymmetry. (Continued on next page)

(Continued from previous page)

**Conclusions:**

“RodCellJ” is freely available to the community as a package of several ImageJ plugins to simultaneously analyze the behavior of a large number of rod-shaped cells in an extensive manner. The integration of different image-processing techniques in a single package, as well as the development of novel algorithms does not only allow to speed up the analysis with respect to the usage of existing tools, but also accounts for higher accuracy. Its utility was demonstrated on both 2D and 3D static and dynamic images to study the septation initiation network of the yeast Schizosaccharomyces pombe. More generally, it can be used in any kind of biological context where fluorescent-protein labeled structures need to be analyzed in rod-shaped cells.

**Availability:**

RodCellJ is freely available under http://bigwww.epfl.ch/algorithms.html.

## Background

Common biological image-analysis tasks typically involve some sort of cell or protein analysis. It is becoming increasingly apparent that spatial control of protein function plays a central role in many aspects of the life of an organism or an individual cell, influencing its development, proliferation, migration or communication between cells. To analyze spatial regulation, image-processing techniques are needed to detect and track fluorescently-tagged proteins, and to measure the intensity of the signal at particular locations, as well as its size and shape. Many well-known model organisms such as the fission yeast *Schizosaccharomyces pombe* and the bacterium *Escherichia Coli* are rod-shaped. In this paper we present an image analysis package to characterize motile structures in rod-shaped cells recorded in fluorescence images. We successfully have tested it on synthetic and real data. In the following subsection we briefly describe the biological application that we used to validate the implementation of our image analysis system.

### Biological application: analyzing spindle pole asymmetry in *S. pombe*

Asymmetry is a key feature of many biological processes; for example, it is essential for specifying cell fate during development, and also for maintenance of stem cells in the adult organism [1]. Asymmetric segregation of regulatory molecules is also important in simple, single celled organisms, such as yeasts; for example, the correct pattern of mating type switching in *S. cerevisiae* requires the sequestration of an RNA in the daughter cell [2]. The fission yeast *S. pombe* has proved to be an excellent model for the study of cell division, including the final step of the cell cycle, cytokinesis. The Septation Initiation Network (SIN) is a key regulator of cytokinesis (reviewed in [3]). SIN proteins associate with the poles of the mitotic spindle (SPBs) via a scaffold of three coiled-coil proteins. In the absence of SIN signaling, cytokinesis does not occur, and cells become multinucleated. In contrast, if SIN signaling is deregulated, cells undergo multiple rounds of septum formation and cytokinesis is uncoupled from its dependency on other cell cycle events. Some SIN proteins distribute asymmetrically on the SPBs during mitosis [4-6], which is thought to be important for regulation of SIN activity [7-9], (reviewed in [10]). We have applied our image-analysis system to characterize spindle pole asymmetry in *S.pombe*.

### Requirements for the image analysis

The task of screening images of cell populations and tracking the SPBs poses several problems. First, all the rod-shaped cells of interest in the images need to be segmented simultaneously, regardless of their orientation. Second, the image quality is inconsistent with respect to parameters such as contrast and noise level. The third problem to be solved is to identify the structures of interest (SOI). In order to analyze the signal intensities of SIN proteins associated with the two SPBs during mitosis, the two SPBs must be located and tracked individually. Since the signal associated with one of the SPBs approaches the threshold of detection at the end of mitosis, we use a second, SPB-associated protein whose fluorescence intensity does not vary significantly throughout mitosis as a reference. Therefore, the software must track two structures (the two SPBs) in two different channels (red, for the reference protein, green, for the SIN protein of interest). Finally, since the orientation of the mitotic spindle is variable with respect to the long axis of the cell, particularly in the earliest stages of mitosis, the software has been designed to analyze both 2D and 3D image stacks.

### Related work and state of the art

Cell segmentation and protein tracking are widely studied subjects in biological image processing. Usually the two tasks are tackled separately. For cell segmentation well-known models range from machine learning-like algorithms [11] to level set methods [12] and texture analysis [13]. Recent developments in wavelet theory have also contributed to the topic of cell segmentation [14,15] as well as to the research in object tracking [16]. Other popular models include graph cuts [17] or approaches based on probabilities [18]. The implementation of our image-analysis system, called “RodCellJ” has several advantages over existing tools for cell segmentation and tracking of fluorescently labeled structures. Our model combines the task of cell segmentation and protein tracking into a single algorithm and introduces several steps to increase the robustness of the tracking routine. For example, unlike other Ťtwo-stepŤ tracking algorithms that first detect structures and then link them through a minimizing criterion [19,20], we implemented a dynamic programming approach to reconstruct the globally optimal track that ensures robustness with respect to intensity variations and can be applied to data with a high noise level. Our segmentation approach has been optimized for the analysis of rod-shaped cells [21], by designing a novel parametric active contour model [22]. We also use of the fact that the structures to be tracked remain inside the cell to increase the robustness of the analysis. Finally, since an asymmetry of signal intensities of only two-fold can be considered significant, we implemented an algorithm that estimates the local shape of the structure being studied, taking into account spatial correlations, as well as background fluorescence, image noise and image quality.

By combining multiple analysis tasks into a single package, RodCellJ provides an additional benefit to the user: the use of different software to solve bio-image analysis tasks often results in file format conversion issues that force people to do a substantial part of the analysis by hand, which can be time consuming and makes it more difficult to take full advantage of computational accuracy and speed. Though the software was designed with a specific task in mind, the algorithm has been implemented in a generic manner to make it useful to a wider community interested in rod-shaped cells. RodCellJ is easy to run and is freely available as an ImageJ [23] and Fiji [24] plugin. ImageJ is a popular open source and public-license image analysis software that can be easily extended by additional plugins. The fact that it is open-source facilitates the reproduction of results through its transparent processing pipeline. To our knowledge, there exists no software yet, implementing these image analysis tasks into a single algorithm, allowing an efficient and rapid analysis of migrating structures inside rod-shaped cells in the possible context of high-throughput screening.

## Results and discussion

### Algorithm

The goals in designing the algorithm were as follows; first, the algorithm for signal detection should account for background noise and background fluorescence within the cells, in order to provide a level of accuracy that cannot be achieved when evaluating the images by eye. Second, after image-analysis, we wished to visualize the result of detected asymmetry of SPB-associated SIN proteins as kymographs representing the SPB migration with respect to each other, as well as the movement of SPBs with respect to the cell. Third, the relevant parameters of the cell being analyzed, such as its width, length, orientation angle, location of its extremities points, must also be extracted. Finally, information about the fluorescently labeled structures of interest, such as their exact position and intensity on each frame should be displayed in a table and saved in a tab-delimited text file for further statistical evaluations (e.g. with Excel, Matlab, etc.). Though RodCellJ is capable of analyzing a time-lapse series of image stacks, if an image representing a single time-point is used as the input, RodCellJ, only does the cell segmentation and protein identification including the calculation of cell and protein specific parameters. The implementation of the algorithm that leads to the final result is generated in a sequential manner, allowing the user to verify and edit intermediate results in a semi-automatic and user-friendly way to guarantee robustness and accuracy.

#### Modularisation

**Figure 1:**
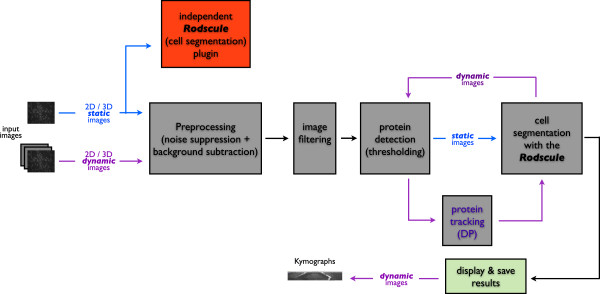
**Modularization of the RodCellJ-package.** 2D and 3D static and dynamic images can be analyzed with the software. The core modules of the software are the protein detection-, the DP- and the Rodscule-modules. Quantitative cell and protein related parameters can be calculated and saved. In the case of dynamic images kymographs can be established. The plugin for cell segmentation implementing the Rodscule is also separately available.

**Figure 2:**
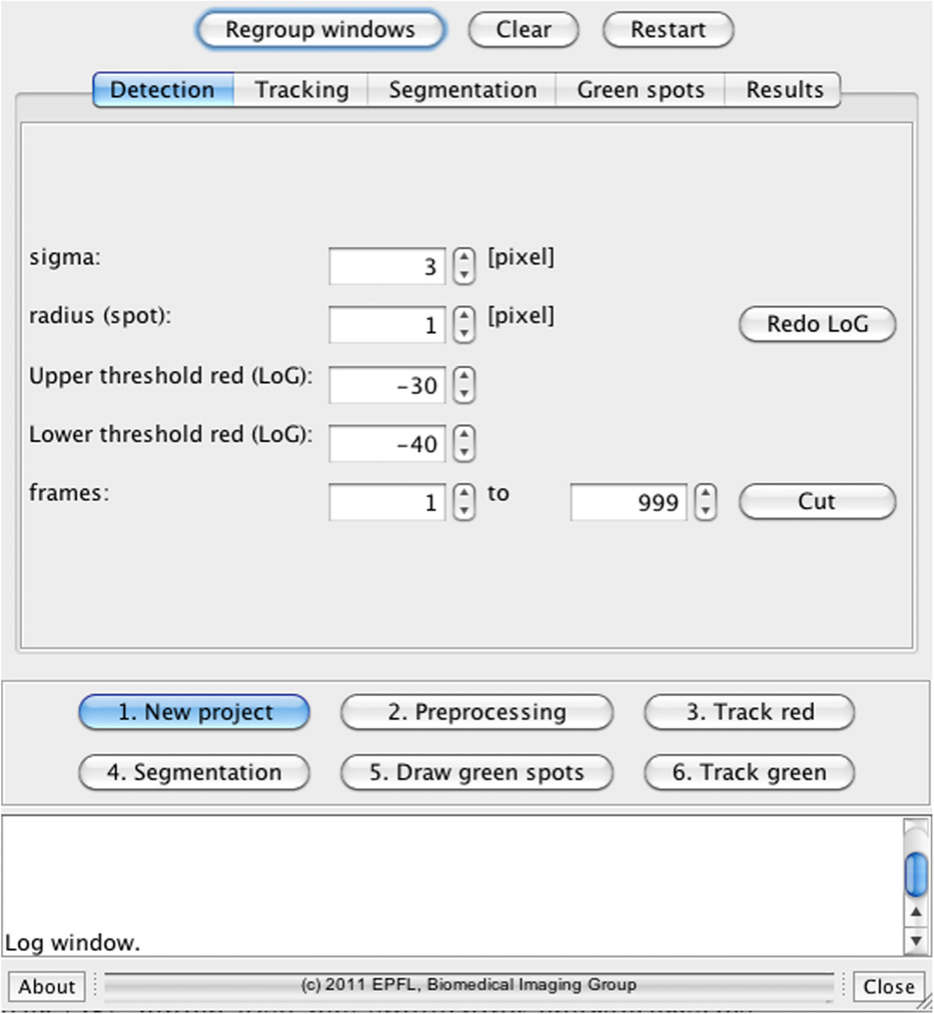
**GUI.** Front panel of the graphical user interface for the 3D-dynamic case.

The different sub-tasks that are carried out by the software to yield quantification of the fluorescent-protein labeled, SPB-associated SIN protein signals are implemented in a modular way (Figure 1). Their execution is handled in an interactive way through a graphical user interface (GUI) as shown in Figure 2. Its purpose is to guide the user through the different steps allowing for the verification of intermediate results giving the possibility of editing them. In the following sections the different modules and their underlying algorithm are explained in detail. We first describe the tracking algorithm, then the segmentation method, since that corresponds to the order of their implementation.

#### Structure-associated fluorescent-protein detection (static images) and tracking (dynamic images)

In this section we describe a robust algorithm to determine the spatio-temporal trajectory of structures (in this case, SPBs) in very noisy dynamic image sequences, such as time-lapse movies. Since the SPBs appear as spots in the image, we will refer to them as such in the following discussion. First, the images are processed with a spot enhancing filter, the Laplacian of Gaussian (LoG) [25], which also has smoothing characteristics enabling noise and background removal. The LoG has the advantage that it is fully characterized by only one parameter (*σ*), which is directly related to the size of the spot that has to be detected. In addition to the noise level of the image a spot does not have the same intensity throughout the image sequence. In some images the spot is no longer clearly distinguishable from the noise. To overcome these difficulties we track the spots with a dynamic programming algorithm [25], a robust method that is able to deal with time-varying signals in noisy conditions. For this purpose we take advantage of the aspects that characterize the behavior of the spots. The biological context allows us to make the following assumptions: 

• the spots remain in the region within a (segmented) cell.

• the movement of the spots is limited by a few pixels from frame to frame.

• the starting point is defined by the spot detection in the first image of the time-lapse movie.

#### Dynamic programming

We can reformulate our problem of spot tracking in terms of finding the optimal path for a spot throughout the image sequences taking into account the characteristics of the data mentioned above, which in turn is the same as finding the optimal path between a pair of vertices in an acyclic weighted graph. In this context “optimality” refers to the assumptions made above.

We define a vertex as Υ_*i*_ and the path from Υ_*i*_ to Υ_*j*_ as Ω_*ij*_. We observe that if the optimal path Ω_*kl*_ passes through Υ_*p*_, then the two sub-paths Ω_*kp*_ and Ω_*pl*_ also must be optimal. Therefore, the problem satisfies Bellman’s principle of optimality, which states that the globally optimum solution includes no suboptimal (local) solution. Hence, we can solve our problem by dynamic programming (DP) [26].

For an analytical formulation of the problem we first need to state the following conditions: 

• A path Ω_*ij*_ has cost C(i,j).

• The graph contains *n* vertices numbered 0,1,…,n−1 and has an edge from Υ_*i*_ to Υ_*j*_ only if *i*<*j* (causality condition).

• Υ_0_ is the source vertex and Υ_*n*−1_ is the destination (which is unknown).

If we define G(x) the cost of the optimal path from Υ_0_ to Υ_*x*_, then

(1) $$G(x)=\left\{\begin{array}{ll} 0 & \text{if}\;x=0\\ \underset{0\leq j<x}{\text{min}}\{G(j)+C(j,x)\} & \text{if}\;1\leq x\leq n-1 \end{array}\right)$$

Thus, for every possible spot $S_{k}$, with k∈{0,…,K} on the last frame *n*−1, we end up with a path Ω_0*n*−1,*k*_ with cost $G_{k}(n-1)$. Note that *K* is the number of possible spots on the last frame. The overall optimal path then is given by

(2) $$\Omega_{\text{opt}}=\Omega_{0n-1,\omega }$$

where *ω* verifies

(3) $$G_{\omega }=\underset{0\leq k\leq K}{\text{min}}\{G_{k}(n-1)\}$$

**Figure 3:**
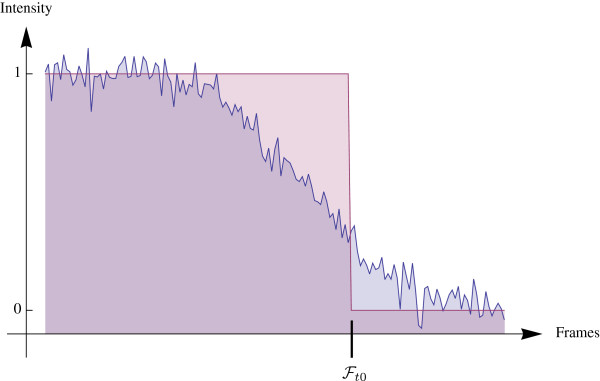
**Detection of loss of fluorescence of the tracked particle.** The blue curve represents the decay of fluorescence of a protein. The frame where fluorescence is lost is represented as the intersection between the red and the blue curve. (Theoretically it corresponds to the time point where the correlation between the blue and the red curve is maximal).

The cost function is defined as follows:

(4) $$C(i,j)=\overset{Q}{\underset{q}{\sum }}\lambda_{q}\;f_{q}(i,j)$$

where the *λ*_*q*_ are weighting factors that can be adjusted through the GUI and the *f*_*q*_(*i*,*j*) are parameters relevant to the image data such as intensity, intensity variation, distance of migration as well as possible directional persistence if required. For example the cost function penalizes heavily a spot that is outside of the segmented cell that surrounds the starting location. Therefore, without imposing absolute restrictions the optimal path through the noisy data can be found. An additional criterion that we have to be aware of is that the spots are not necessarily visible until the last frame of the time-lapse movie, because in the usual asymmetric case the signal strength of one of the two spots decreases significantly at some point in the time trajectory. Therefore, we implemented an additional global criterion to detect the frame $F_{t0}$, where it was last visible. This consists of calculating a threshold that depends on the noise and background level of the image sequence, below which a spot is no longer distinguishable from noise (Figure 3).

#### Estimating the signal intensity of the spot

In the case where they appear as spots in images, a common method that is used to estimate signal intensities of labeled proteins is to take the extreme value that is detected in a certain neighborhood (e.g. local minima/maxima). This method may yield inaccurate values because it does not account for the influence of local background, noise or the shape of the structure being analyzed. A much more precise method for fluorescence particle intensity estimation is to take into account the point spread function (PSF) of the microscope. According to [27,28] an unbiased estimation of the PSF can be obtained by modeling it with a 2D Gaussian function taking into account the diffraction limit for the microscope and the pixel size of the image. In our algorithm we implemented a model that accounts for these issues (the default microscope related parameters can be specified by the user through the GUI of the plugin). After the detection of the local extrema we fit a 2D Gaussian curve to it including a possible offset for noise/background estimation (5) and a rotation angle *θ*.

(5) $$A*e^{-\left[\left\{\frac{\text{co}s^{2}\theta }{2\sigma_{x}^{2}}+\frac{\text{si}n^{2}\theta }{2\sigma_{y}^{2}}\right\}{\left(x-\mu_{x}\right)}^{2}+2\left\{-\frac{\text{sin}\left(2\theta \right)}{4\sigma_{x}^{2}}+\frac{\text{sin}\left(2\theta \right)}{4\sigma_{y}^{2}}\right\}\left(x-\mu_{x}\right)\left(y-\mu_{y}\right)+\left\{\frac{\text{si}n^{2}\theta }{2\sigma_{x}^{2}}+\frac{\text{co}s^{2}\theta }{2\sigma_{y}^{2}}\right\}{\left(y-\mu_{y}\right)}^{2}\right]}+B$$

where the value of *B* captures the background and noise, *x*,*y* stand for the two dimensions of the image plane and *μ*_*x*_,*μ*_*y*_ and *σ*_*x*_,*σ*_*y*_ are the center of the 2D Gaussian and its variance respectively. The initial variances to initialize the optimization algorithm can directly be estimated by dividing the value obtained for the diffraction limit by the corresponding value of the pixel size. Therefore, the actual value estimated for the protein intensity corresponds to *A*. The rotation parameter *θ* describes the rotation with respect to the Cartesian coordinate system (i.e. if *θ*=0 we end up with the general expression of a 2D Gaussian, where the variances point in *x* and *y* direction). Using this method we additionally circumvent the drawback of the discretization of the image in terms of intensities and spatial coordinates to obtain much more precise values than in the case of doing only integer calculations. For the estimation of the parameters of the Gaussian we use the Levenberg-Marquardt algorithm [29].

#### Cell segmentation with an active contour model

The method that we use for cell segmentation strongly depends on the nature of the image data. In order to use our algorithm the cells need to be rod-shaped and immobile. There is no restriction with respect to the size and orientation of the cells (even in the same image cells with different sizes can be segmented simultaneously as long as they are rod-shaped). The cells can have arbitrary and different orientations within the same image and the number of cells that has to be segmented in an experiment is unlimited, as long as they do not overlap each other in the image. The algorithm requires that the area in the image that is delimited by the cell membrane (i.e. the inside of the cell) should have a different intensity than the background of the image. To test our software package we used *S. pombe* cells expressing cdc7p-GFP. This protein associates with the SPBs during mitosis. There is also a significant cytoplasmic signal, which allows us to identify cells against the background. The protein is excluded from the nucleus, generating a dark “hole” in each cell (two in late mitotic cells).

#### Active contour model a.k.a. “snake”

Models that do not exploit shape information such as watershed or region growing approaches may produce oversegmentation due to the noise level and possible intensity inhomogeneities within the cell background. The problem cannot be solved by a a simple conversion to a binary image followed by “filling the holes” because the cell’s cytoplasmic fluorescence intensities are not sufficiently consistent to enclose a convex set. Taking into account these considerations, we decided that an active contour model suits our needs best. Therefore, we took advantage of the cells’ rod-shape to formulate a parametric shape model which ensures robust segmentation.

**Figure 4:**
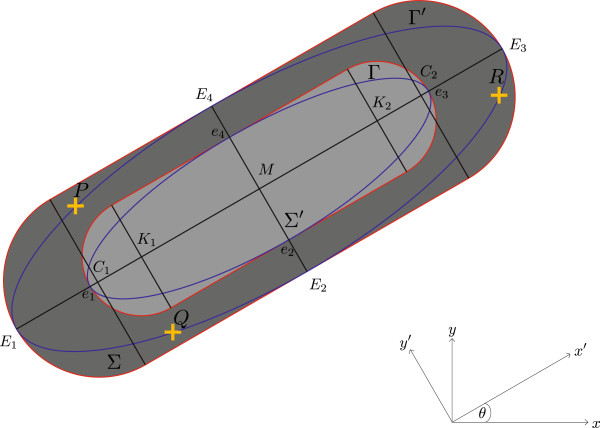
**The Rodscule.** The inner and outer rod, Σ^′^,Σ, (red) are defined by an inner and an outer ellipse, Γ^′^,Γ, (blue). The 3 points *P*,*Q*,*R* (yellow) define the ellipse and, hence, the Rodscule. *M* is the barycenter, *K*_1_ and *K*_2_ the centers of the semi-circles of the inner rod, *C*1,*C*2 the centers of the semi-circles of the outer rod. *e*_1_,*e*_2_,*e*_3_,*e*_4_ and *e*_1_,*e*_2_,*e*_3_,*e*_4_ are the extremal points of the inner and outer ellipse respectively. They define the common points of each ellipse with its corresponding rod, where it is inscribed.

Since the position of the cells remains fixed throughout the image stack we first calculate the (average) z-projection of the image stack, where the z-axis is perpendicular to the image plane. This reduces noise. Afterwards a contrast enhancement is performed. For the actual segmentation of the cells we designed an active contour model, in the literature often called “snake”, in the form of a rod shaped structure. Following the annotation of the snakes described in [30,31] we call it the “Rodscule”. The Rodscule is a surface snake, which means that a certain energy function can be associated to it that depends on the image data enclosed by it. It consists of an inner rod Σ^′^ and an outer rod *σ* (Figure 4).

The Rodscule optimizes an energy term that should be minimal when the contrast between the image data averaged over |Σ^′^| and |Σ|∖|Σ^′^| is maximal. Here, |Σ| is the area of the outer rod *σ* and |Σ^′^| is the area of the inner rod Σ^′^. The energy term is given by (6), where the directions of *x* and *y* define the Cartesian coordinate system. It is important that $\left|\Sigma^{\prime }|=\frac{1}{2}|\Sigma \right|$. Thus, none of the two energy sub-terms overweights if we apply the Rodscule over a region where everywhere the intensity is constant (in that case we have $E_{R}=0$). For simplicity, we want the inner rod to have the same orientation as the outer rod. To find the minimum of $E_{R}$ we use a conjugate gradient-based method (the derivation of the gradient $\nabla E_{R}$ can be found in the Additional file 1).

(6) $$E_{R}=\frac{1}{\left|\Sigma \right|}\left(\int_{\Sigma \setminus \Sigma^{\prime }}f(x,y)\text{dxdy}-\int_{\Sigma^{\prime }}f(x,y)\text{dxdy}\right)$$

To minimize the computational cost we imposed some restrictions on the parametrization of our snake. First, we used as few parameters as possible, with the additional condition that they should be independent of each other. Second, we want the impact on the area of a changing of a parameter by a small *δX* to be the same for every parameter. This excludes the possibility of parametrizing the Rodscule by two points *P*,*Q* representing the centers of the two semi-circles and a third point *R* where the distance $\bar{\text{PR}}$ determines the radius of the semi-circles (Figure 4).

From the Ovuscule [30] we know that an ellipse can be parametrized by three arbitrary points that define a triangle. These three points belong to the border of the ellipse. Since our rod shape can be defined by an ellipse where the four extremal points of the ellipse belong to the border of the rod shape, this means that the rodscule can be defined by exactly the same three points which define the ellipse and, hence, the Ovuscule (Figure 4). The two ellipses and the two rod shapes all have the same barycenter. The complete derivation of the expression of $E_{R}(P,Q,R)$ as well as a detailed description of the construction and implementation of the Rodscule can be found in the Additional file 1.

### Validation

#### Cell segmentation on synthetic data simulating noisy conditions

**Figure 5:**
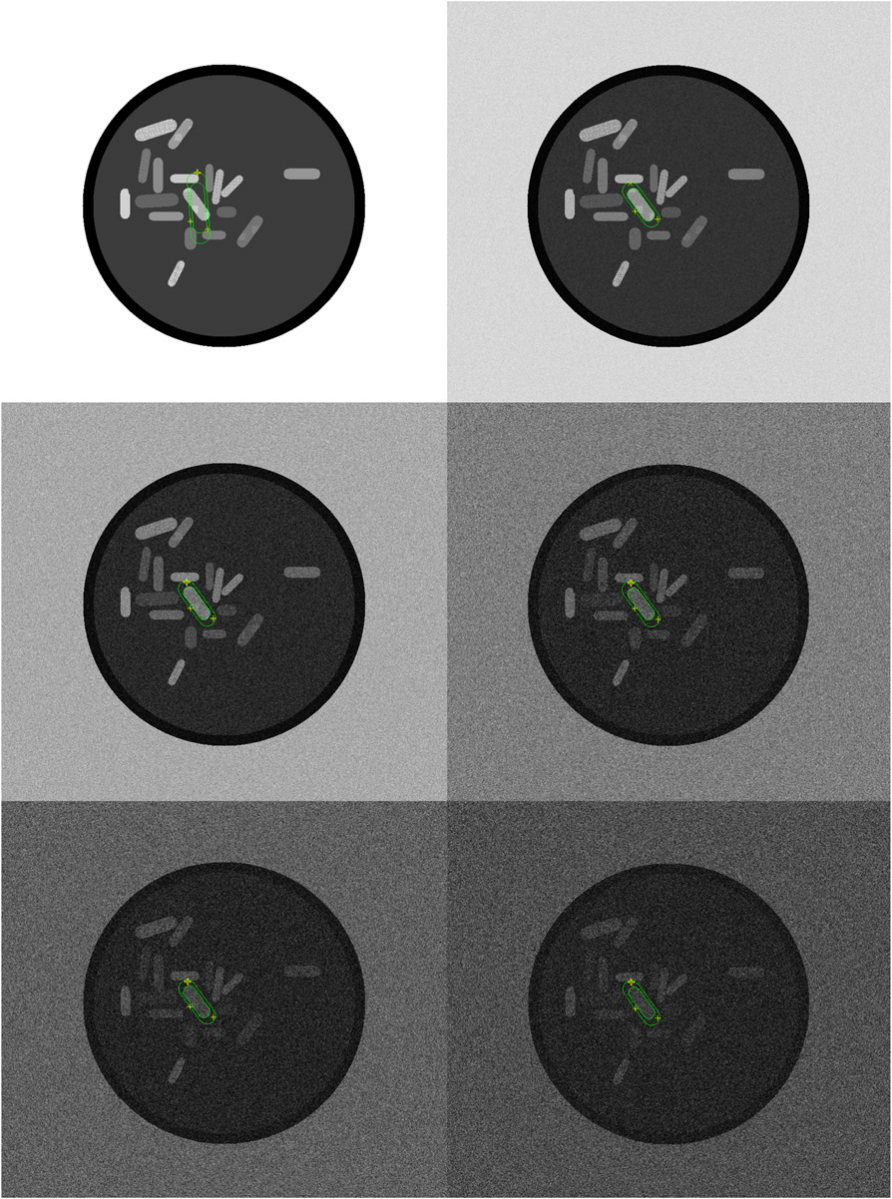
**Robustness of the Rodscule in the prescence of noise.** The top-left image shows the initial configuration of the rodscule before triggering the optimization for cell segmentation. In the remaining images the additive white noise was successively augmented. Its standard deviations are {10, 30, 60, 90, 120} and the corresponding SNR is equal to {26.2, 16.8, 11.1, 7.6, 5.3} dB.

**Figure 6:**
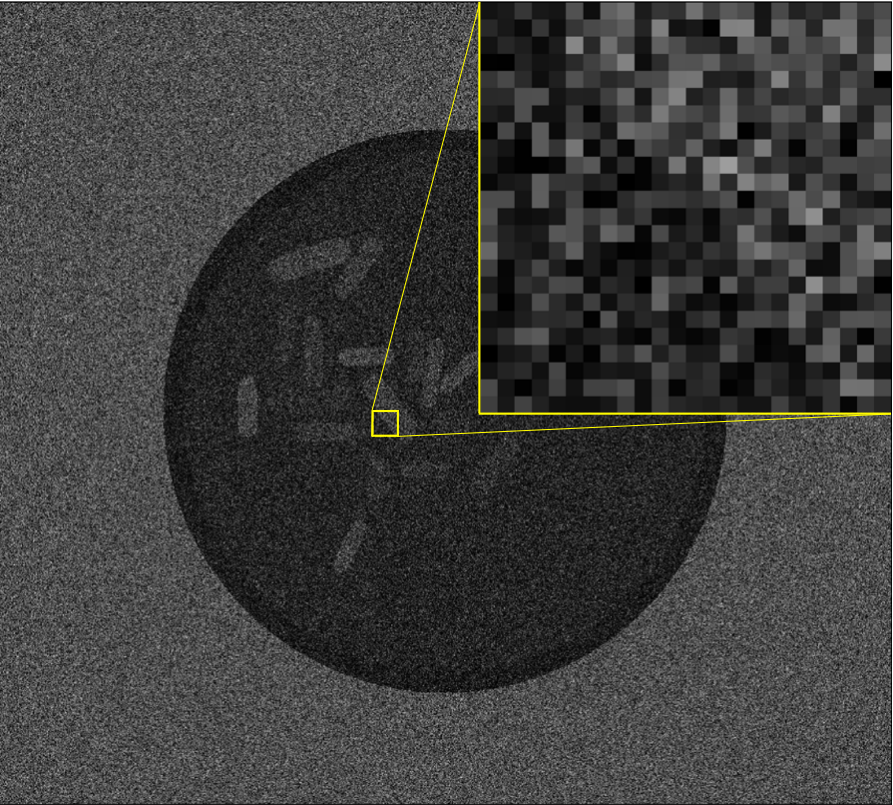
**Close-up of a boundary region between an artificially created cell and its background.** The phantom image that is shown was corrupted by withe noise (std =120, SNR =5.3).

**Figure 7:**
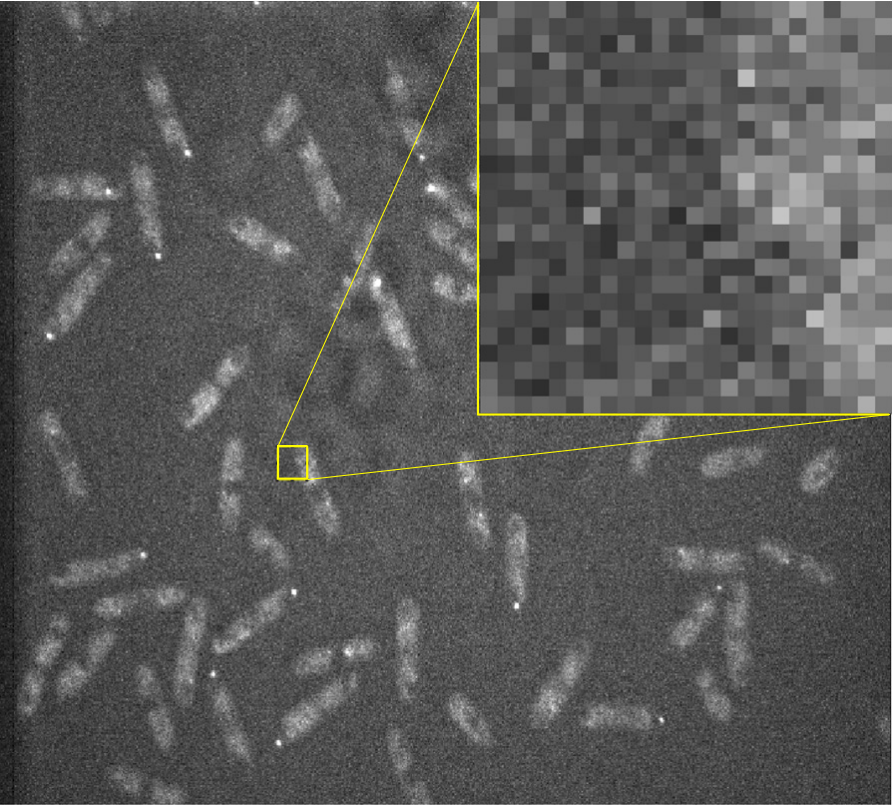
Close-up of a boundary region between a real cell and its background.

In this experiment we validate our active contour model on synthetic data. We successively augmented the presence of additive Gaussian white noise in the artificially created phantom image shown in Figure 5, while running our algorithm on it. In the top left image (Figure 5), the initial configuration of the Rodscule is shown. It remains the same throughout the experiment. In the remaining 5 images the standard deviations (std) of the noise were increased. They correspond to {10, 30, 60, 90, 120} (with respect to an 8-bit gray scale image, where the pixels take values between 0 and 255) and the resulting decrease in the signal-to-noise ratio (SNR) for the same remaining 5 images is equal to {26.2, 16.8, 11.1, 7.6, 5.3} dB. In all 5 images the Rodscule found the correct segmentation only through the optimization process demonstrating the robustness of the algorithm with respect to the presence of photo-metric noise. Figure 6 represents the same image as the bottom-right of Figure 5 (std =120, SNR =5.3 dB). It additionally shows a close-up of a boundary region between the segmented artificial cell and its background to emphasize the advantage of the Rodscule over segmenting by human eye. While it seems very difficult to find the ground truth segmentation in this image manually, the Rodscule does so due to the optimal way it minimizes the corresponding energy function. Figure 7 shows an analogous example of a real cell.

#### Segmentation of yeast cells

**Figure 8:**
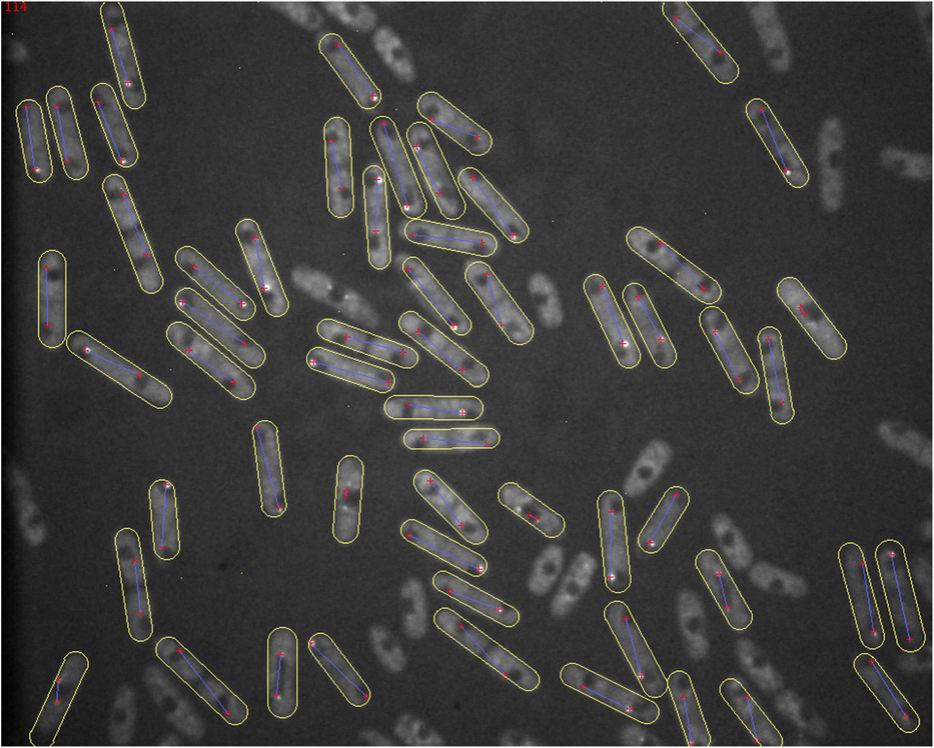
**Simultaneous segmentation of yeast cells.** The two red spots in each cell are proteins attached to the spindle pole bodies that were detected before the segmentation. They are used to initialize the approximate orientation of the snake. The blue line connecting them shows the initial orientation. The cells are outlined by a yellow contour. The cells that are not segmented did not express fluorescent protein attached to the SPB and therefore did not show any POI.

We demonstrate the utility of the segmentation algorithm on real data using images of the fission yeast *S. pombe*. They are typically rod-shaped but the length of the cell can vary within the same experiment. In contrast to the previous experiment, we now want to segment many cells simultaneously. Since the outcome of the optimization algorithm for cell segmentation depends on the location of initialization, the two spots (SPBs) were detected before segmentation. Since they approximately define the longitudinal axis of the cell (they are oriented towards the two poles of the cell) we can use them to initialize the Rodscules. Figure 8 shows the result of such an experiment. In an experiment where 212 cells had to be segmented we segmented 184 cells correctly, yielding a rate of 87% of true positives. In the case where the segmentation fails, the user can reinitialize the segmentation by one mouse-click and subsequent dragging of the mouse to change the initialization position. The editing of a cell takes about 3 seconds.

#### Protein tracking

For the evaluation of the protein tracking algorithm implemented with the DP routine we refer to the results of prior work (Sage et al.) [25], where we have shown that with our tracking algorithm we are able to trace particles in images where the peak signal-to-noise ratio (PSNR) on average is as low as 0 dB. The PSNR is defined as

**Figure 9:**
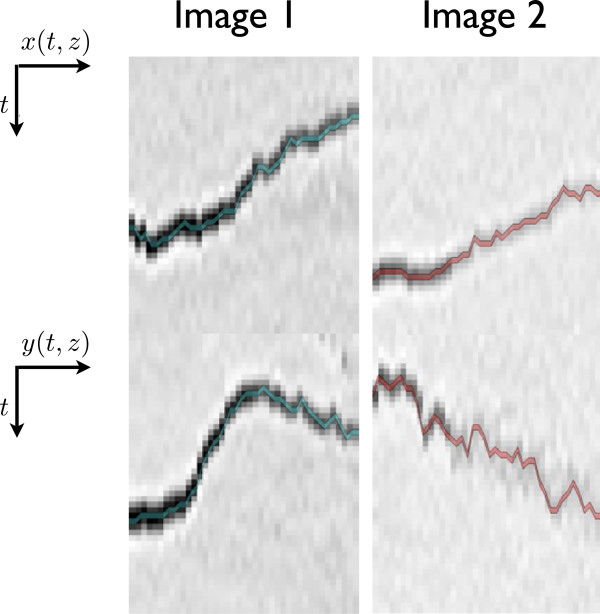
**Protein (particle) tracking in different SNR conditions.** The images originate from a 42-frame time-lapse movie. For both images (left/right column) the evolution of *x* and *y* respectively in time are shown. The left column shows a spot where the fluorescence decreases in time. *x*, *y* and *z* correspond to the usual 3D Cartesian coordinates.

PSNR=20log(A/σ)

with *σ* being the noise variance and *A* the amplitude of the Gaussian-shaped spot. Figure 9 illustrates an example of a result obtained with our tracking routine. It shows a comparison between two particles, one yielding a high SNR, whereas the fluorescence of the second spot decreases with time (decreasing SNR).

#### Spot signal intensity estimation

**Figure 10:**
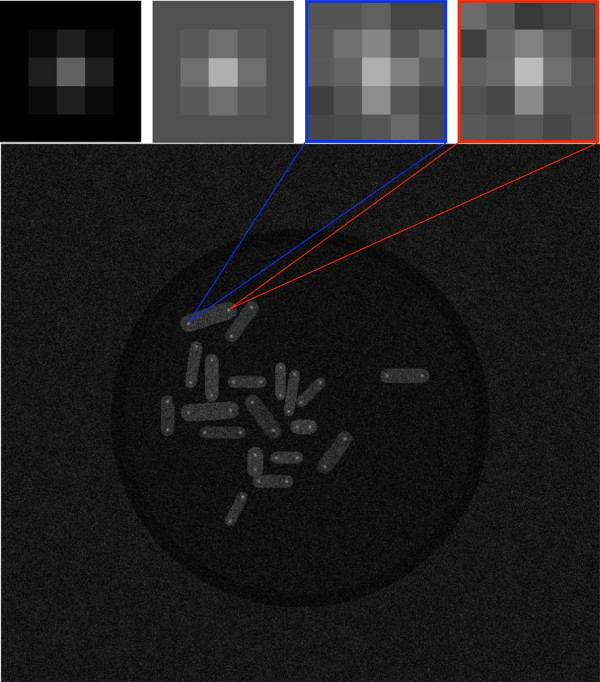
**Protein intensity estimation by a 2D Gaussian kernel.** Top, from left to right: 2D Gaussian kernel without offset (specified by Table 1); 2D Gaussian kernel including offset (specified by Table 2); 2D Gaussian kernel after adding additive Gaussian white noise (std = 15, enframed in blue); 2D Gaussian kernel after adding additive Gaussian white noise (std = 15, enframed in red); Bottom image: synthetically created phantom image, where additive Gaussian white noise was added (std = 15).

**Figure 11:**
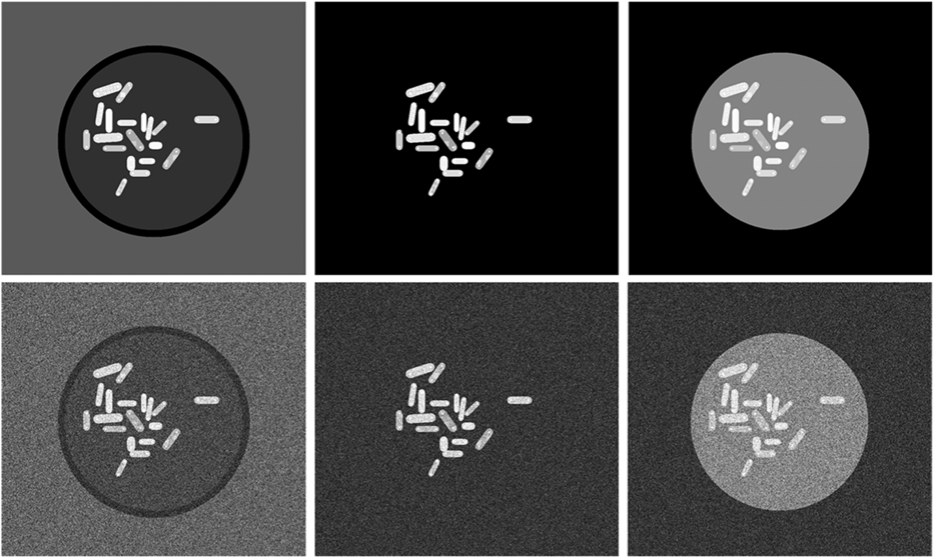
**Protein intensity estimation on different background intensities.** In each of the 6 images the intensities of the cells and proteins remain the same. Note that each cell contains two bright dots at its poles, which represent the SPBs. However, the image background and the intensity of the simulated petri dish (disk containing the cells) vary from left to right. The bottom row shows the images of the top row with additive random Gaussian white noise (std = 15).

For the evaluation of our routine for spot intensity estimation, in a first step we compare it to two existing standard techniques commonly used by biologists. For this purpose we created artificial images showing cells, each containing two simulated spots (SPBs) (Figure 10, bottom). The shape and intensities of the spindle pole bodies (i.e. the two white spots located towards the poles of each cell) are calculated using our 2D Gaussian approximation to estimate the PSF described above. Table 1 shows the discretized Gaussian kernel that we used to carry out this experiment. In order to test the robustness of our algorithm we added an arbitrarily chosen offset value to the kernel of the 8-bit test image (i.e. intensity values are between 0 and 255) as shown in Table 2. Additive Gaussian white noise (std =15) was then added to the whole image. Figure 10 (top row) shows an example of such a corrupted Gaussian kernel used to simulate such proteins of interest. In our synthetic images we gave the cells significant cytoplasmic fluorescence. However, our spot intensity estimation can also be applied when this is not the case, since the intensity of the signal in the vicinity of the spot does not influence the algorithm. To further demonstrate the robustness of the algorithm we chose three different image/petri dish backgrounds for testing as shown in Figure 11. The two techniques that we used to compare our method with, are the well-known “rolling ball” algorithm [32] and the maximum intensity technique, where the maximum intensity value in a well-defined neighborhood (e.g. 5 ×5 pixels) of the protein is evaluated. Table 3 summarizes the results of the comparison. It shows the mean absolute error, $e_{\text{mean}-\text{abs}}=\overset{n}{\underset{k=1}{\sum }}|I_{k}-I_{\text{real}}|$, as well as the standard deviations obtained with the three different methods. Here *k* is the index that runs over all detected spots *n*=38, *i*_*k*_ is the intensity obtained with respect to the method applied and *I*_*real*_ is the actual intensity that should be detected (i.e. 95.9719, the maximum of the Gaussian described in Table 1).

**Table 1:** 5 × 5 2D Gaussian kernel approximation

0.0074	0.2584	0.8439	0.2584	0.0074
0.2584	8.9997	29.3891	8.9997	0.2584
0.8439	29.3891	95.9719	29.3891	0.8439
0.2584	8.9997	29.3891	8.9997	0.2584
0.0074	0.2584	0.8439	0.2584	0.0074

The table shows the values used to approximate a 2D Gaussian with standard deviation 0.65. The values are scaled to correspond to an 8 bit image (i.e. between 0 and 255).

**Table 2:** 5 ×5 2D Gaussian kernel approximation with an offset of 80

80.0074	80.2584	80.8439	80.2584	80.0074
80.2584	88.9997	109.3891	88.9997	80.2584
80.8439	109.3891	175.9719	109.3891	80.8439
80.2584	88.9997	109.3891	88.9997	80.2584
80.0074	80.2584	80.8439	80.2584	80.0074

The table contains the values of the 2D Gaussian described in Table 1, where a constant offset, *b*=80, was added.

**Table 3:** Comparison of different protein intensity estimation algorithms

	**Figure 11 - left**	**Figure11 - middle**	**Figure 11- right**
**mean error Gauss. fit.**	11.5199	10.8913	12.6662
**mean error max. int.**	81.7386	81.6860	82.5018
**mean error roll. ball**	6.4447	76.7913	64.9228
**std Gauss. fit.**	13.8731	14.2601	16.7261
**std max. int.**	14.1286	14.0699	15.7938
**std roll. ball**	5.2605	14.0471	15.9506

The table shows the performance of the Gaussian fitting algorithm compared to the rolling ball algorithm as well as to the maximum intensity detection.

Looking at the values shown in Table 3, it becomes evident that our algorithm is very robust with respect to noise and, additionally, is quite independent from the background of the image. Furthermore, because the algorithm approximates the PSF of the microscope in theory it is totally independent from the vicinity of the 2D Gaussian and only depends on the values enclosed by it.

Even though there is great variability between the three test images shown in Figure 11 (bottom row), our algorithm only introduces little variability in terms of spot intensity estimation. The percentage error is $e_{\text{PSF}-\text{fit}}=0.14=\frac{\left(11.5199+10.8913+12.6662\right)}{256}$, whereas with the rolling ball algorithm we obtain *e*_*rol*.−*ball*_=0.58 and with the maximum intensity method *e*_*max*.−*int*._=0.96. Analyzing these results we notice that the two latter methods are highly dependent on the background values. Furthermore the maximum intensity method is also very susceptible to the different kind of noise in the image. The rolling ball algorithm is less influenced by noise, however it still remains unsuitable if the background of the image is not uniform or if the image contains other features than the background, such as in our test images, where the cell has significant background fluorescence and the petri dish is also visible in the image.

#### Comparison of manual and automatic evaluation

A test protocol was designed in order to reflect the manual evaluation conditions in a realistic manner. For this purpose four human observers (*o*∈{1,2,3,4}) evaluated the 3 test images (*i*∈{1,2,3}) shown in Figure 11 (bottom) manually using the standard image analysis tools of ImageJ. The goal was to measure the intensities of a previously defined number of spots in each image. The images were resized using bilinear interpolation to obtain a 10x magnification in order to facilitate the observers’ task. The observers were free to use the available tools (e.g. image zoom, contrast adjustment, etc.) knowing that the procedure was meant to simulate real work conditions (i.e. it was their choice how to manage the trade-off between time constraint and accuracy of the result). The time of evaluation per image was also measured. The whole procedure was repeated with the same images with a background subtraction corresponding to the rolling ball algorithm [32]. In total all observers evaluated each image three times (*r*∈{1,2,3}). We define the interobserver and intraobserver variability as

$$\begin{array}{lll} V_{\text{inter},s} & =\frac{1}{18\cdot P}\overset{3}{\underset{r=1}{\sum }}\overset{4}{\underset{i=1}{\sum }}\overset{4}{\underset{j=i+1}{\sum }}\overset{P}{\underset{p=1}{\sum }}|x_{s,i,r}^{p}-x_{s,j,r}^{p}|\; & \;\\ V_{\text{intra},s} & =\frac{1}{12\cdot P}\overset{4}{\underset{o=1}{\sum }}\overset{3}{\underset{i=1}{\sum }}\overset{3}{\underset{j=i+1}{\sum }}\overset{P}{\underset{p=1}{\sum }}|x_{s,o,i}^{p}-x_{s,o,j}^{p}|\; & \; \end{array}$$

where *s*∈{*orig*,*bckSub*} refers to the original image or the image where the background was subtracted respectively and *p* runs over all the spots, *P*, considered in an image. ***x***∈{(*x*_1_,*x*_2_),*I*}, where *I*=*I*(*x*_1_,*x*_2_) stands for the intensity and (*x*_1_,*x*_2_) represents the spatial coordinate of a point in an image, implying that the interobserver and intraobserver variability can be measured with respect to the evaluated intensity values itself, *I*(*x*_1_,*x*_2_), or their spatial coordinate (*x*_1_,*x*_2_). The subscripts of ***x*** correspond to ***x***_*s*,*o*,*r*_. The variabilities with respect to intensity estimation are shown in Table 4. We notice that the very high interobserver variability makes it difficult to obtain reproducible results. Although the mean intensity values calculated over all series of the 6 test images (Figure 11, bottom; 3 times without and 3 times with background subtraction) were 105.19 and 76.58 respectively (the true intensity is 95.97, all values are on a 8-bit scale, i.e. in the range [0,255]) the high standard deviations of 20.33 and 58.90 again suggest that the manual method is unreliable and not well suited to obtain comparable results with respect to a general norm that is independent of the operator or user. These results are clearly in favor of our method, which yields stable results with low standard deviations (see Table 3). Furthermore, due to the local convergence of our algorithm there is no interoperator variability when evaluating the results obtained by different users. The average time required by the users to measure the intensity of 38 spots was 1 min 37 sec, whereas (depending on the computer used) RodCellJ can evaluate the intensities about 100 times faster (c.f. section “Computational aspects”).

**Table 4:** Variability of the manual evaluation of spot intensity estimation

	**Original image**	**Background**
		**subtracted image**
**mean intervariability**	36.4	23.71
**maximum intervariability**	37.98	28.28
**std intervariability**	1.54	3.23
*mean intravariability*	0.84	0.28
*maximum intravariability*	1.03	0.47
*std intravariability*	0.16	0.13
variability RodCellJ	0.0	-
std RodCellJ	0.0	-

“original image” refers to the images in Figure 11 (bottom), whereas “background subtracted image” refers to the same images but with subtracted background. The results for interobserver variability are shown in bold font and for the intraobserver variability in italic respectively. Because of the nature of our algorithm, RodCellJ produces zero variability, hence guaranteeing reproducible results.

#### Kymographs

**Figure 12:**
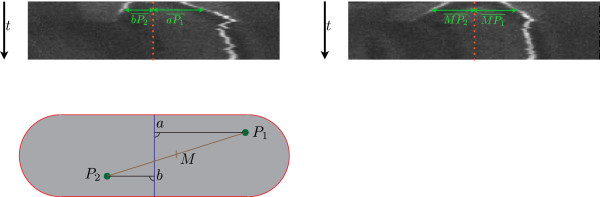
**Kymographs.** Upper left: kymograph representing the movement of the particles with respect to the cell. The dotted red line corresponds to the blue line in the lower left figure, which represents the line going through the center of the rod shape. Hence, this kymograph shows the migration of the proteins compared to the cell center line. Upper right: kymograph showing the movement of the proteins with respect to each other. As indicated by the scheme in the lower left part of the figure, the distances between the proteins and the center line (red dotted) are always equal. It serves to highlight differences in fluorescence. lower left: scheme explaining the different kinds of kymographs.

Once the spots have been tracked, two different kymographs can be established. The first kymograph (Figure 12, upper left) shows the movement of the spots with respect to the cell center (i.e. the barycenter of the Rodscule), whereas the second plots the movement of the spots with respect to each other (Figure 12, upper right). An illustration of these results is provided in Figure 12.

#### Computational aspects

RodCellJ is developed to run on multiprocessor architectures. Several tracking and segmentation algorithms can therefore be run in parallel. There is no limit on the number of cells that can be segmented or the total number of spots that can be tracked per session. On a 2.8 Ghz Intel i7 Quad-Core processor with 16GB SDRAM on average (evaluating 48 cells, 83 tracked spots) it took 971 milliseconds to segment one cell and 823 milliseconds to track one spot through a time-lapse movie containing 50 frames. Fitting the spot shape with the PSF approximation algorithm takes about 36 milliseconds per spot.

## Conclusions

We have presented a new image analysis system to fully characterize the protein analysis in rod-shaped cells. The image analysis system was implemented as an ImageJ/Fiji plugin called RodCellJ. It is able to handle 2D or 3D static and dynamic images. It includes new and robust state-of-the art algorithms to semi-automatically segment rod-shaped cells and detect and track up to four spots in two different channels located within the cells. A novel algorithm to accurately estimate signal intensities independent of their shape and background, which approximates the microscope’s point spread function was also presented. The software outputs several cell- and protein specific parameters. In the case of dynamic image analysis two different kymographs that represent spot migration can be displayed and saved. We successfully demonstrated the utility of this tool to measure the asymmetry of the signal produced by an SPB-associated, GFP-labeled signal transduction protein in *S. Pombe*. The rapidity and efficacy of this tool will allow it to be used for screening large numbers of mutant strains to study their effects upon SIN regulation. Though we have developed this software for the analysis of SPB behaviour during mitosis in fission yeast, it is applicable to tracking other large structures in the cell, for example nuclei. It could also be applied to track other fluorescently-labeled complexes that adopt a spot-like morphology in the cycle.cell.

## Methods

### Cell lines and microscopy

The strains used in this study were obtained from crosses between 2 strains: cdc7(ura4+)EGFP, ura4-D18, leu1-32 h+ and pcp1(ura4+)mCherry ura4-D18, leu1-32 h- to obtain a double mutant carrying both tagged alleles. Cells were grown in yeast extract medium to early exponential phase (exponentially growing culture) and centrifugal elutriation was used to isolate small G2 cells. Cells were concentrated by filtration, and after 1 hour recovery time, the cells were imaged using a Plan-S-Apo 60x N.A. 1.42 objective lens mounted on a Perki-Elmer spinning disk confocal microscope. The culture was sampled for imaging during both the first and second mitoses after elutriation. Images were exported and their parameters were assessed using RodCellJ.

## Abbreviations

SIN: Septation initiation network; SPB: Spindle pole body; GUI: Graphical user interface; LoG: Laplacian of Gaussian; STD: Standard deviation; SNR: Signal-to-noise ratio; PSNR: Peak signal-to-noise ratio; dB: decibel.

## Competing interests

The authors declare that they have no competing interests.

## Authors’ contributions

DSc designed, wrote and implemented the algorithm and contributed significantly to the study design and the draft writing. DSa contributed to the study design, algorithm writing and critical revision of the manuscript. MU contributed to critical revision of the manuscript and the design of the active contour model (Rodscule). PW carried out the cell cultivation and cell imaging experiments and contributed to the draft writing and the validation of RodCellJ. VS participated in the project coordination, study design and manuscript revision. AC contributed to manual cell parameter evaluation. IX contributed to the study design. All authors read and approved the final manuscript.

## Supplementary Material

Additional file 1 **The Rodscule.** Additional file 1 is a pdf containing a complete and detailed descriptioin of the active contour model, named “The Rodscule”, that was implemented in RodCellJ as a model for cell segmentation. It contains its mathematical description and the mathematical derivation of the gradient needed for the optimization algorithm. Additionally a description of its implementation is provided, explaining the issues that had to be solved when discretizing the continuously defined active contour. Click here for file
