# Supplementary material for: A 2D/3D image analysis system to track fluorescently labeled structures in rod-shaped cells: application to measure spindle pole asymmetry during mitosis
Source: Cell Div. 2013 Apr 27;8:6. doi: 10.1186/1747-1028-8-6 (PMC3693874; doi:10.1186/1747-1028-8-6)
Supplement: Additional file 1 — The Rodscule. Additional file 1 is a pdf containing a complete and detailed descriptioin of the active contour model, named “The Rodscule”, that was implemented in RodCellJ as a model for cell segmentation. It contains its mathematical description and the mathematical derivation of the gradient needed for the optimization algorithm. Additionally a description of its implementation is provided, explaining the issues that had to be solved when discretizing the continuously defined active contour. [file 1747-1028-8-6-S1.pdf]

0.1 The Rodscule

0.1.1 Energy

The rodscule is a surface snake, and therefore its energy depends on the image data enclosed by it. It consists of an inner rod Σ' and an outer rod Σ as depicted in Figure 1.

The rodscule optimizes an energy term that should be minimal when the contrast between the image data averaged over $|\Sigma'|$ and $|\Sigma| \setminus |\Sigma'|$ is maximal. Here, $|\Sigma|$ is the area of the outer rod Σ and $|\Sigma'|$ is the area of the inner rod Σ'. The energy is given by (1), where the directions of x and y define the cartesian coordinate system. It is important that $|\Sigma'| = \frac{1}{2}|\Sigma|$. Thus, none of the two energy subterms overweights if we apply the rodscule over a region where everywhere the intensity is constant. (In that case we have $\mathcal{E}_{\mathcal{R}} = 0$). Besides, to keep things simple, we want the inner rod to have the same orientation as the outer rod. To find the minimum of $\mathcal{E}_{\mathcal{R}}$ we use the conjugate gradient-based method. Therefore, we need to calculate the gradient $\nabla(\mathcal{E}_{\mathcal{R}})$ with respect to its defining parameters.

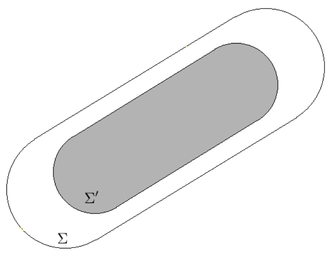

Figure 1: *The inner rod Σ' (gray) and the outer rod Σ (white)*

$$\mathcal{E}_{\mathcal{R}} = \frac{1}{|\Sigma|}\left(\int_{\Sigma\setminus\Sigma'} f(x,y)dxdy - \int_{\Sigma'} f(x,y)dxdy\right) \tag{1}$$

0.1.2 Parametrization

To maintain the computational calculations as simple as possible we impose the following restrictions regarding the parametrization.

- The rodscule should be parametrized by as few parameters as possible.
- The parameters should be independent from each other.
- The impact on the area of a changing of a parameter by a small δX should be the same for every parameter.

Note that the last of the three points mentioned above excludes the possibility of parametrizing the rodscule by two points P, Q representing the centers

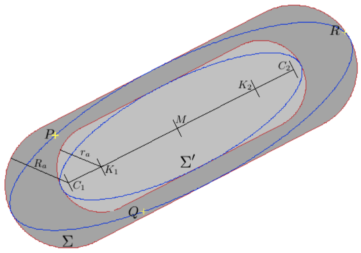

Figure 2: *The inner and outer rod (red) defined by an inner and an outer ellipse (blue). The 3 points P, Q, R (yellow) define the ellipse and, hence, the rodscule. M is the barycenter, K_1 and K_2 the centers of the semi-circles of the inner rod, C_1, C_2 the centers of the semi-circles of the outer rod, r_a the radius of the inner semi-circles and R_a the radius of the outer semi-circles.*

of the two semi-circles and a third point R where the distance $\overline{PR}$ determines the radius of the semi-circles. However, we still want a solution that parametrizes the snake by only three points.

0.1.3 Solution

From the ovuscule [3] we know that an ellipse can be parametrized by three aribtrary points that define a triangle. These three points belong to the border of the ellipse. Since our rod shape can be defined by an ellipse where the four extremal points of the ellipse belong to the border of the rod shape, this means that the rodscule can be defined by exactly the same three points which define the ellipse and, hence, the ovuscule (Figure 2). The two ellipses and the two rod shapes all have the same barycenter. We provide the complete derivation of the expression of $\mathcal{E}_{\mathcal{R}}(P, Q, R)$ in Appendix A.

0.1.4 Optimization

For the optimization process we use the conjugate gradient-based method that requires the calculation of the gradient of the engergy $\nabla(\mathcal{E}_{\mathcal{R}})$. (Obviously, this requires $\mathcal{E}_{\mathcal{R}}$ to be differentiable with respect to all of its parameters). In the following we provide an analytical expression of the partial derivatives of $\mathcal{E}_{\mathcal{R}}$.

If we define

$$I = \int_{\Sigma\setminus\Sigma'} f(x, y)dxdy - \int_{\Sigma'} f(x, y)dxdy = I_1 - I_2 \tag{2}$$

then (1) can be expressed as

$$\mathcal{E}_{\mathcal{R}} = \frac{I}{|\Sigma|} \tag{3}$$

It follows that for any $Z \in \{P, Q, R\}$:

$$\begin{aligned}\frac{\partial \mathcal{E}_{\mathcal{R}}}{\partial Z} &= \frac{\frac{\partial I}{\partial Z}|\Sigma| - I\frac{\partial|\Sigma|}{\partial Z}}{|\Sigma|^2} = \frac{1}{|\Sigma|}\frac{\partial I}{\partial Z} - \frac{I}{|\Sigma|^2}\frac{\partial|\Sigma|}{\partial Z} \\ &= \frac{1}{|\Sigma|}\frac{\partial I}{\partial Z} - \frac{\mathcal{E}_{\mathcal{R}}}{|\Sigma|}\frac{\partial|\Sigma|}{\partial Z}\end{aligned} \tag{4}$$

If we calculate I_1 and I_2 only for the interior of their domains of definition (i.e. none of its points belongs to a border), then

$$\frac{\partial I}{\partial Z} = 0 \tag{5}$$

and (4) simplifies to

$$\frac{\partial \mathcal{E}_{\mathcal{R}}}{\partial Z} = -\frac{\mathcal{E}_{\mathcal{R}}}{|\Sigma|}\frac{\partial|\Sigma|}{\partial Z} \tag{6}$$

(6) will become particularly useful for implementing the rodscule (see section: 0.1.6 Implementation).

0.1.5 Domains of transitions

We can express (1) as

$$\mathcal{E}_{\mathcal{R}} = \frac{1}{|\Sigma|}\int_{\mathbb{R}^2} \xi(X - M) f(X) dxdy \tag{7}$$

where $X = (x, y)$, M is the common barycenter of the rod shapes and

$$\xi(X - M) = \begin{cases} 1 & \text{if} \quad X \in \Sigma \setminus \Sigma' \\ -1 & \text{if} \quad X \in \Sigma' \\ 0 & \text{else} \end{cases} \tag{8}$$

Equation (8) emphasizes that along the borders $\partial\Sigma'$ of Σ' and $\partial\Sigma$ of Σ conflicting situation may arise. For this reason and following the argumentation in [3] we define two extended transition zones: one at the frontier between Σ' and Σ and another at the frontier of Σ and its complement (i.e. its exterior). That way we can define ξ as a continuous function in $\mathbb{R}$. For this purpose we define g as the line segment between K_1 and K_2 and G the line segment between C_1 and C_2 (see Figure 2). Recall that r_a is the radius of the inner semi-circles and R_a is the radius of the outer semi-circles. Every point $X \in \mathbb{R}^2$ can now be classified according to the definitions of the following 5 domains:

$$\mathcal{D}_1 = \{X : \quad \overline{gX} < r_a - \frac{1}{\sqrt{2}}\} \tag{9}$$

$$\mathcal{D}_2 = \{X \notin \mathcal{D}_1 : \quad \overline{gX} < r_a + \frac{1}{\sqrt{2}}\} \tag{10}$$

$$\mathcal{D}_3 = \{X \notin (\mathcal{D}_1 \cup \mathcal{D}_2) : \quad \overline{GX} < R_a - 1\} \tag{11}$$

$$\mathcal{D}_4 = \{X \notin (\mathcal{D}_1 \cup \mathcal{D}_2 \cup \mathcal{D}_3) : \quad \overline{GX} < R_a + 1\} \tag{12}$$

$$\mathcal{D}_5 = \{X \in \mathbb{R}^2 \setminus \bigcup_{k=1}^{4} D_k\} \tag{13}$$

Now we are able to define ξ by

$$\xi(X-M) = \begin{cases} -1 & \text{if} \quad X \in \mathcal{D}_1 \\ \sqrt{2}(\overline{gX} - r_a) & \text{if} \quad X \in \mathcal{D}_2 \\ 1 & \text{if} \quad X \in \mathcal{D}_3 \\ \frac{1}{2}(R_a + 1 - \overline{GX}) & \text{if} \quad X \in \mathcal{D}_4 \\ 0 & \text{if} \quad X \in \mathcal{D}_5 \end{cases} \tag{14}$$

Figure 3 illustrates the definition of these five sets in the case of an image given by its discrete samples.

0.1.6 Implementation

An image can only theoretically be defined by its continuous values $f(X)$ in (7), whereas in practice it is given by its discrete samples $f[\tau]$. Therefore, if we want to implement $\mathcal{E}_{\mathcal{R}}$(P,Q,R) we need to discretize it by

$$\mathcal{E}_{\mathcal{R}}[P,Q,R] = \frac{1}{|\Sigma|} \sum_{\tau \in \mathbb{R}^2} \xi(\tau - M) f[\tau] \tag{15}$$

For the optimization process we calculate the gradient for each of the domains specified by (9), (10), (11), (12) and (13) individually. Analytical expressions are given in Appendix B. However, because already the simplest out of total 24 partial derivatives (4 domains, 6 partial derivatives each) yields 230 pages of Mathematica output we disclaim to append them in their full expression. Instead, we content ourselves with a numerical solution when implementing it. Therefore, for each of the three points P, Q, R we calculate the energy by moving one of them by a ΔX sequentially in the four directions of the cartesian coordinate system while keeping the remaining two points fixed. For each position of the rodscule this procedure yields 15 different energy values (4 values for each point plus the initial position of each point). Finally, we chose the configuration that yields the lowest of these 15

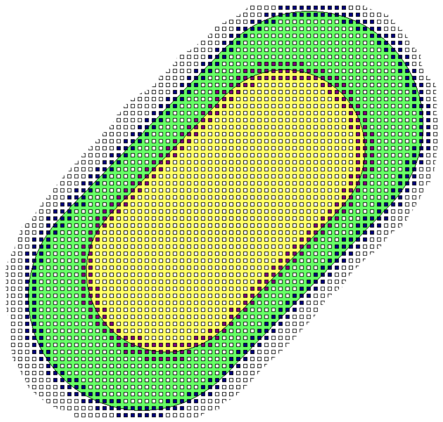

Figure 3: $\mathcal{D}_1$: *white squares in yellow region,* $\mathcal{D}_2$: *violet squares,* $\mathcal{D}_3$: *white squares in green region,* $\mathcal{D}_4$: *blue squares,* $\mathcal{D}_5$: *white squares outside of the outer rod.*

energies as the new configuration. If no lower energy than the one yielded by the initial configuration is found the snake stops looking for prey.

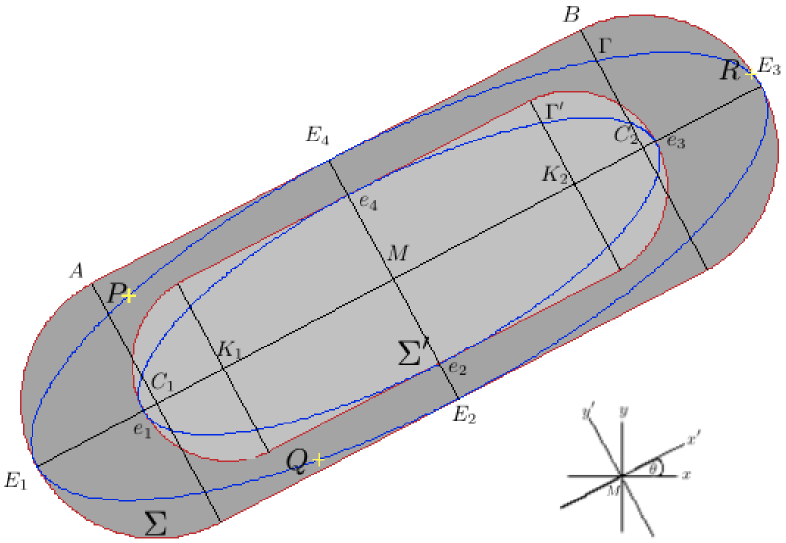

Figure 4: *Inner and outer rod (red) with inner and outer ellipse (blue), as well as P, Q, R (yellow) and other relevant points necessary for the derivation of the energy function. The coordinate system indicates the orientation of the rodscule. Note that it is centered on M.*

explanation of variables

P, Q, R: points defining the rodscule
M: barycenter
K_1, K_2: centers of semi-circles of the inner rod
C_1, C_2: centers of semi-circles of the outer rod
$e_1, \ldots, e_4$: extremal points of inner ellipse
$E_1, \ldots, E_4$: extremal points of outer ellipse
Σ: outer rod
Σ': inner rod
Γ: outer ellipse
Γ': inner ellipse
θ: angle that defines the orientation of the rodscule

A: upper left corner of rectangle of outer rod shape.
B: upper right corner of rectangle of outer rod shape.

x', y' define the coordinate system that matches the orientation of the rod-scule, whereas x, y define the cartesian coordinate system.

A Derivation of the energy function

A.1 Characteristics of the ellipse

A.1.1 Parametric form

According to [3] the following characteristics of the ellipse can be obtained given P, Q, R:

if we define $\partial\Gamma$ as the border of Γ and $\partial\Gamma'$ as the border of Γ', then

$$\forall X \in \partial\Gamma : X(\theta) = M + c \cdot cos\theta + s \cdot sin\theta \tag{16}$$

and

$$\forall X' \in \partial\Gamma' : X'(\theta) = M + \frac{1}{\sqrt{2}}(c \cdot cos\theta + s \cdot sin\theta) \tag{17}$$

where $\theta \in [-\pi, \pi]$, $c = \frac{2P-Q-R}{3}$, $s = \frac{Q-R}{3}$, $M = \frac{1}{3}(P + Q + R)$

A.1.2 Extremal points

The four extremal points are given by

$$\theta_k = \frac{1}{2}arctan(\frac{u}{v}) + (k-1)\frac{\pi}{2} \tag{18}$$

where $k \in \{1, \ldots, 4\}$,

$u = Q_x(2P_x - Q_x) + Q_y(2P_y - Q_y) - R_x(2P_x - R_x) - R_y(2P_y - R_y)$

$v = \sqrt{3}(P_x^2 - 3M_x^2 + P_y^2 - 3M_y^2 + 2(Q_xR_x + Q_yR_y))$

Combining (16) and (18) we get

$E_k = X(\theta_k)$ for the 4 extremal points of the outer ellipse.

Combining (17) and (18) we get

$e_k = X'(\theta_k)$ for the 4 extremal points of the inner ellipse.

A.2 Characteristics of the rod shape

A.2.1 Orientation

The orientation is given by

$$\theta = arctan(\frac{E_{3y} - M_y}{E_{3x} - M_x}) \tag{19}$$

A.2.2 Radii of semi-circles

We define R_a the radius of the semi-circles of the outer rod shape and r_a the radius of the semi-circles of the inner rod shape. They are given by

$$R_a = \overline{E_4M} \tag{20}$$

$$r_a = \frac{1}{\sqrt{2}}R_a = \frac{1}{\sqrt{2}}\overline{E_4M} \tag{21}$$

A.2.3 Centers of semi-circles

The centers of the semi-circles of the outer rod shape are C_1, C_2 and the ones of the inner rod shape are K_1, K_2. The are given by

$$C_{1x} = E_{1x} + R_a cos\theta \tag{22}$$

$$C_{1y} = E_{1y} + R_a sin\theta \tag{23}$$

$$C_{2x} = E_{3x} - R_a cos\theta \tag{24}$$

$$C_{2y} = E_{3y} - R_a sin\theta \tag{25}$$

$$K_{1x} = e_{1x} + r_a cos\theta \tag{26}$$

$$K_{1y} = e_{1y} + r_a sin\theta \tag{27}$$

$$K_{2x} = e_{3x} - r_a cos\theta \tag{28}$$

$$K_{2y} = e_{3y} - r_a sin\theta \tag{29}$$

A.2.4 Corners

The two corner points A and B are needed for the definition of $|\Sigma|$. A is given by

$$A_x = C_{1x} + R_a cos(\theta + \frac{\pi}{2}) \tag{30}$$

$$A_y = C_{1y} + R_a sin(\theta + \frac{\pi}{2}) \tag{31}$$

whereas B is given by

$$B_x = C_{2x} + R_a cos(\theta + \frac{\pi}{2}) \tag{32}$$

$$B_y = C_{2y} + R_a sin(\theta + \frac{\pi}{2}) \tag{33}$$

A.2.5 Area

The area $|\Sigma|$ of the outer rod shape is given by

$$|\Sigma| = 2R_a\overline{AB} + \pi R_a^2 \tag{34}$$

A.3 Energy function

$$\begin{aligned} \mathcal{E}_{\mathcal{R}} &= \frac{1}{|\Sigma|}\left(\int_{\Sigma\setminus\Sigma'} f(x,y)dx'dy' - \int_{\Sigma'} f(x,y)dx'dy'\right) \\ &= \frac{1}{|\Sigma|}\left(\int_{\Sigma} f(x,y)dx'dy' - 2\int_{\Sigma'} f(x,y)dx'dy'\right) \end{aligned} \tag{35}$$

We need to express x as $x(x')$ and y as $y(y')$.

Hence,

$$x = M_x + x'cos(\theta) \tag{36}$$

$$y = M_y + y'sin(\theta) \tag{37}$$

Combining (35), (36) and (37) we obtain

$$\begin{aligned}\mathcal{E}_{\mathcal{R}} &= \frac{1}{|\Sigma|}\left(\int_{\Sigma} f(M_x + x'cos(\theta), M_y + y'sin(\theta))dx'dy'\right. \\ &\left. -2\int_{\Sigma'} f(M_x + x'cos(\theta), M_y + y'sin(\theta))dx'dy'\right) \\ &= \frac{1}{|\Sigma|}(\mathcal{E}_{\mathcal{R}\Sigma} - 2\mathcal{E}_{\mathcal{R}\Sigma'})\end{aligned} \tag{38}$$

where $\mathcal{E}_{\mathcal{R}\Sigma}$ and $\mathcal{E}_{\mathcal{R}\Sigma'}$ are given by

$$\begin{aligned}\mathcal{E}_{\mathcal{R}\Sigma} &= \int_{C_{1y}-R_a}^{C_{1y}+R_a} \int_{C_{1x}}^{C_{2x}} f(M_x + x'cos(\theta), M_y + y'sin(\theta))dx'dy' \\ &+ \int_{\theta+\frac{\pi}{2}}^{\theta+\frac{3\pi}{2}} \int_{0}^{R_a} f(C_{1x} + rcos\phi, C_{1y} + rsin\phi)rdrd\phi \\ &+ \int_{\theta-\frac{\pi}{2}}^{\theta+\frac{\pi}{2}} \int_{0}^{R_a} f(C_{2x} + rcos\phi, C_{2y} + rsin\phi)rdrd\phi\end{aligned} \tag{39}$$

$$\begin{aligned}\mathcal{E}_{\mathcal{R}\Sigma'} &= \int_{K_{1y}-r_a}^{K_{1y}+r_a} \int_{K_{1x}}^{K_{2x}} f(M_x + x'cos(\theta), M_y + y'sin(\theta))dx'dy' \\ &+ \int_{\theta+\frac{\pi}{2}}^{\theta+\frac{3\pi}{2}} \int_{0}^{r_a} f(K_{1x} + rcos\phi, K_{1y} + rsin\phi)rdrd\phi \\ &+ \int_{\theta-\frac{\pi}{2}}^{\theta+\frac{\pi}{2}} \int_{0}^{r_a} f(K_{2x} + rcos\phi, K_{2y} + rsin\phi)rdrd\phi\end{aligned} \tag{40}$$

B Gradient of the energy function

The energy function is given by (7)

$$\mathcal{E}_{\mathcal{R}} = \frac{1}{|\Sigma|}\int_{\mathbb{R}^2} \xi(X - M)f(X)dxdy \tag{7}$$

where $\xi(X - M)$ is given by (14)

$$\xi(X-M) = \begin{cases} -1 & \text{if} \quad X \in \mathcal{D}_1 \\ \sqrt{2}(\overline{gX} - r_a) & \text{if} \quad X \in \mathcal{D}_2 \\ 1 & \text{if} \quad X \in \mathcal{D}_3 \\ \frac{1}{2}(R_a + 1 - \overline{GX}) & \text{if} \quad X \in \mathcal{D}_4 \\ 0 & \text{if} \quad X \in \mathcal{D}_5 \end{cases} \tag{14}$$

For each of the domains $\mathcal{D}_k$, $k \in \{1, \ldots, 5\}$ we are going to give an expression for the gradient. It is obvious that for $\mathcal{D}_5$ we have $\nabla \mathcal{E}_{\mathcal{R}} = 0$. For domains $\mathcal{D}_1$ and $\mathcal{D}_3$ we can apply (6)

$$\frac{\partial \mathcal{E}_{\mathcal{R}}}{\partial Z} = -\frac{\mathcal{E}_{\mathcal{R}}}{|\Sigma|} \frac{\partial |\Sigma|}{\partial Z} \tag{6}$$

whereas for domains $\mathcal{D}_2$ and $\mathcal{D}_4$ we need to calculate the whole expression of (4)

$$\frac{\partial \mathcal{E}_{\mathcal{R}}}{\partial Z} = \frac{1}{|\Sigma|} \frac{\partial I}{\partial Z} - \frac{\mathcal{E}_{\mathcal{R}}}{|\Sigma|} \frac{\partial |\Sigma|}{\partial Z} \tag{4}$$

Formally, the gradient of $\mathcal{E}_{\mathcal{R}\mathcal{D}_k}$ is given by

$$\nabla\{\mathcal{E}_{\mathcal{R}\mathcal{D}_k}\}(P, Q, R) = \nabla\{\mathcal{E}_{\mathcal{R}\mathcal{D}_k}\}(P_x, P_y, Q_x, Q_y, R_x, R_y) =$$

$$= \left(\frac{\partial \mathcal{E}_{\mathcal{R}\mathcal{D}_k}}{\partial P_x}, \frac{\partial \mathcal{E}_{\mathcal{R}\mathcal{D}_k}}{\partial P_y}, \frac{\partial \mathcal{E}_{\mathcal{R}\mathcal{D}_k}}{\partial Q_x}, \frac{\partial \mathcal{E}_{\mathcal{R}\mathcal{D}_k}}{\partial Q_y}, \frac{\partial \mathcal{E}_{\mathcal{R}\mathcal{D}_k}}{\partial R_x}, \frac{\partial \mathcal{E}_{\mathcal{R}\mathcal{D}_k}}{\partial R_y} \right) \tag{41}$$

where $k \in \{1, \ldots, 5\}$

The overall gradient $\nabla \mathcal{E}_{\mathcal{R}}$ is given by

$$\nabla \mathcal{E}_{\mathcal{R}} = \sum_{k=0}^{5} \nabla \mathcal{E}_{\mathcal{R}\mathcal{D}_k} \tag{42}$$

B.0.1 Gradient over $\mathcal{D}_1$

From (40) we obtain

$$
\begin{aligned}
\mathcal{E}_{\mathcal{R}\mathcal{D}_1} =& \int_{K_{1y}-(r_a-\frac{1}{\sqrt{2}})}^{K_{1y}+(r_a-\frac{1}{\sqrt{2}})} \int_{K_{1x}}^{K_{2x}} f(M_x + x'cos(\theta), M_y + y'sin(\theta))dx'dy' \\
&+ \int_{\theta+\frac{\pi}{2}}^{\theta+\frac{3\pi}{2}} \int_0^{r_a-\frac{1}{\sqrt{2}}} f(K_{1x} + rcos\phi, K_{1y} + rsin\phi)rdrd\phi \\
&+ \int_{\theta-\frac{\pi}{2}}^{\theta+\frac{\pi}{2}} \int_0^{r_a-\frac{1}{\sqrt{2}}} f(K_{2x} + rcos\phi, K_{2y} + rsin\phi)rdrd\phi
\end{aligned} \tag{43}
$$

According to (6) we can calculate the partial derivatives of $\mathcal{E}_{\mathcal{R}\mathcal{D}_1}$ by

$$
\frac{\partial \mathcal{E}_{\mathcal{R}\mathcal{D}_1}}{\partial Z} = -\frac{\mathcal{E}_{\mathcal{R}\mathcal{D}_1}}{|\Sigma|}\frac{\partial |\Sigma|}{\partial Z} \tag{44}
$$

where $Z \in \{P, Q, R\}$

B.0.2 Gradient over $\mathcal{D}_2$

The energy over $\mathcal{D}_2$ is given by

$$
\begin{aligned}
\mathcal{E}_{\mathcal{R}\mathcal{D}_2} =& \int_{K_{1y}-(r_a+\frac{1}{\sqrt{2}})}^{K_{1y}+(r_a+\frac{1}{\sqrt{2}})} \int_{K_{1x}}^{K_{2x}} f(M_x + x'cos(\theta), M_y + y'sin(\theta))dx'dy' \\
&+ \int_{\theta+\frac{\pi}{2}}^{\theta+\frac{3\pi}{2}} \int_0^{r_a+\frac{1}{\sqrt{2}}} f(K_{1x} + rcos\phi, K_{1y} + rsin\phi)rdrd\phi \\
&+ \int_{\theta-\frac{\pi}{2}}^{\theta+\frac{\pi}{2}} \int_0^{r_a+\frac{1}{\sqrt{2}}} f(K_{2x} + rcos\phi, K_{2y} + rsin\phi)rdrd\phi \\
&- \mathcal{E}_{\mathcal{R}\mathcal{D}_1}
\end{aligned} \tag{45}
$$

Its partial derivatives are given by (4). Thus,

$$
\frac{\partial \mathcal{E}_{\mathcal{R}\mathcal{D}_2}}{\partial Z} = \frac{1}{|\Sigma|}\frac{\partial I}{\partial Z} - \frac{\mathcal{E}_{\mathcal{R}\mathcal{D}_2}}{|\Sigma|}\frac{\partial |\Sigma|}{\partial Z} \tag{46}
$$

B.0.3 Gradients over $\mathcal{D}_3$ and $\mathcal{D}_4$

The gradients over $\mathcal{D}_3$ and $\mathcal{D}_4$ are calculated as in B.0.1 and B.0.2 respectively, where the limits of the integrals must be changed according to the definitions of (11) and (12) and the energies of the excluded domains must be substracted.

References

[1] Andrew Blake and Michael Isard. *Active Contours.* Springer, 1998.

[2] M. Jacob, T. Blu, and M. Unser. Efficient energies and algorithms for parametric snakes. *IEEE Transactions on Image Processing*, 13(9):1231–1244, September 2004.

[3] P. Thévenaz, R. Delgado-Gonzalo, and M. Unser. The ovuscule. *IEEE Transactions on Pattern Analysis and Machine Intelligence*, 33(2):382–393, February 2010.

[4] Mark J. Burge Wilhelm Burger. *Digital Image Processing: an algorithmic introduction using Java.* Springer, 2008.
